# The opposing association of diet and serum sodium with the prevalence of hypertension in the US adult general population: A cross-section study

**DOI:** 10.1097/MD.0000000000045103

**Published:** 2025-10-24

**Authors:** Hongpeng Liu, Jianmei Zhou, Qing Wu, Shanshan Xing

**Affiliations:** a Department of Radiology, Zhejiang Hospital, Hangzhou, China; b Department of Cardiac Rehabilitation, Zhejiang Hospital, Hangzhou, China; c Zhejiang Provincial Key Lab of Geriatrics, Zhejiang Hospital, Hangzhou, China; d Department of Organization and Personnel (Talent Development Office), Zhejiang Hospital, Hangzhou, China.

**Keywords:** cross-sectional study, dietary sodium, hypertension, NHANES, serum sodium

## Abstract

This study was designed to explore the cross-sectional association between dietary and serum sodium levels and the risk of hypertension within the general US population. A total of 15,349 adult participants were obtained from the National Health and Nutrition Examination Survey from 2011 to 2018. Weighted logistic regression analyses were employed to examine the associations between dietary and serum sodium and hypertension. The weighted restricted cubic spline was constructed based on the fully adjusted model to explore the dose–response relationship. Additionally, further stratified analyses were carried out. All data handling and analyses were executed using the “Survey” package in R software (Version 4.4.1). The mean age of the study population was 47.53 ± 0.33 years, with an average body mass index of 29.36 ± 0.12 kg/m^2^. Males accounted for 48.11%, and the weighted prevalence of hypertension was 38.14%. This study uncovered a positive association between the highest quartile of dietary sodium intake and the risk of hypertension among older adults, females, overweight or obese individuals, nonsmokers and nondrinkers, those with low levels of physical activity, and those without cardiovascular diseases. Moreover, a “V”-shaped nonlinear relationship was identified between serum sodium levels and the risk of hypertension among older, sedentary participants. Adopting a low-sodium diet and maintaining serum sodium levels at around 141 mmol/L may confer significant health advantages. Such an approach holds the potential to decrease the risk of hypertension and enhance overall cardiovascular health.

## 1. Introduction

Hypertension, commonly referred to as high blood pressure (BP), is a chronic medical condition characterized by persistently elevated arterial pressure. Nicknamed the “silent killer,” hypertension often presents without symptoms. However, if left untreated, it can lead to severe complications. The continuous force exerted by high BP gradually damages blood vessels and organs, significantly increasing the risk of cardiovascular diseases (CVDs), such as stroke, myocardial infarction, heart failure, and renal failure.^[1]^ According to epidemiological reports, the global prevalence of hypertension was estimated to be 29% in 2025, showing an upward trend.^[2]^ Data from the World Health Organization indicates that noncommunicable diseases, including CVDs and hypertension, account for approximately 41 million deaths annually, constituting 71% of the total annual global deaths.^[3]^ Despite extensive research and public health efforts to reduce its prevalence, hypertension remains a persistent challenge, imposing a substantial burden on the social health system.

Fortunately, hypertension can often be managed or even prevented through lifestyle modifications, such as a healthy diet and regular physical activity.^[4]^ In some cases, medications are essential for achieving effective BP control. Thus, hypertension is regard as the foremost preventable risk factor for CVDs and disability globally. Of importance, sodium modification is a cornerstone of non-pharmacological management of hypertension. Animal experiments, epidemiological studies, and clinical trials have all documented a causal relationship between high salt intake and elevated BP.^[5]^ A recent meta-analysis of 48 randomized clinical trials revealed that an average 42 mmol/d reduction in sodium intake lowered systolic BP by 3.23 mm Hg (95% confidence interval [CI]: 2.41–4.06) and diastolic BP by 2.24 mm Hg (1.61–2.96).^[6]^ However, a substantial body of evidence from both animal and human studies has indicated that the BP elevation in response to salt intake might be more specifically attributable to the anionic component namely chloride ion, rather than sodium.^[7,8]^ Additional, a national dataset study elucidated that the dietary sodium intake level was inversely associated with CVDs mortality.^[9]^ This inverse association raises questions regarding the likelihood of a survival advantage accompanying a lower salt diet.

Serum sodium is closely associated with dietary sodium.^[10]^ Emerging evidence suggests that even subtle changes in plasma sodium might represent a key mechanism underlying the alterations in BP in response to varying salt intake. In the general population, it has reported that low serum sodium was independently associated with an increased risk of cardiovascular mortality.^[11]^ Moreover, many research findings have revealed the adverse consequences of low serum sodium in specific populations. A prospective cohort study demonstrated that lower serum sodium concentration, even within the normal range, was a major risk factor for mortality in Korean elderly adults.^[12]^ Another prospective, hospital-based epidemiological study conducted in China revealed that the serum sodium concentration showed a statistically significant negative association with coronary events and all-cause mortality in subjects with coronary atherosclerosis.^[13]^ BP is the most powerful predictor of stroke and other cardiovascular events. Nevertheless, conflicting results regarding the association between serum sodium and BP have also been reported. Some cross-sectional studies have found a positive association between serum sodium and BP,^[14,15]^ while others have reported on association^[16,17]^ or a negative relationship.^[18]^

Understanding of the relationship between dietary and serum sodium levels and the risk of hypertension is crucial can help refine public health strategies and offer novel insights into the prevention and management of hypertension, particularly for individuals following a low-sodium diet. Therefore, this study utilized the National Health and Nutrition Examination Survey (NHANES) dataset, to explore the distribution of dietary and serum sodium levels within the general population and to investigate their impacts on the risk of hypertension among the adult general population in the United States.

## 2. Methods

### 2.1. Study population

The NHANES adopted a cross-sectional design. It used stratified, multistage probability sampling of the US population to examine health and nutritional status through household interviews, physical examinations, laboratory tests and health-related questionnaires.^[19]^ The survey was approved by the Ethics Review Board of the National Center for Health Statistics in accordance with the Declaration of Helsinki, the approval details are accessible on the web at: https://www.cdc.gov/nchs/nhanes/. All participants supplied written informed permission.

**Figure 1.**
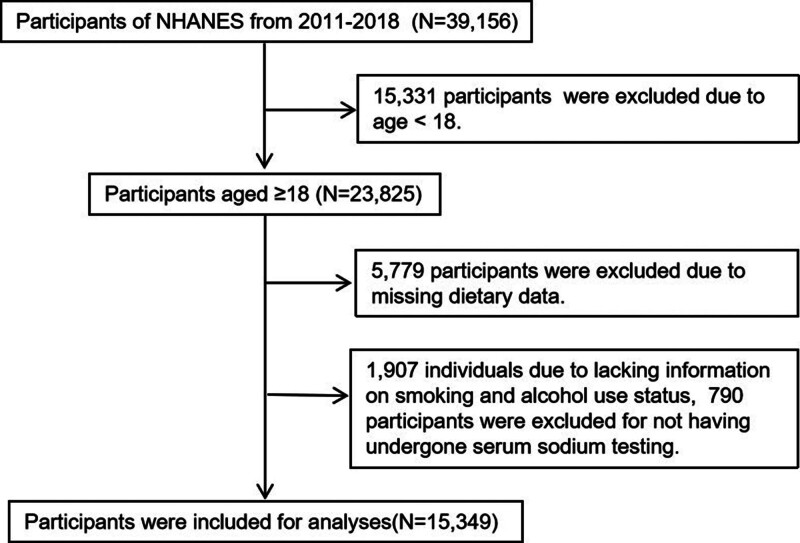
Work flow. NHANES = national health and nutrition examination survey.

This study was based on the demographic, dietary, laboratory, and questionnaire databases of the NHANES. A total of 39,156 participants took part in NHANES from 2011 to 2018. Among them, 23,858 participants were 18 years of age or older. Subsequently, we excluded 5779 individuals owing to missing dietary data and 1907 individuals due to lacking information on smoking and alcohol use status. Additionally, 790 participants were excluded for not having undergone serum sodium testing. Ultimately, 15,349 participants were included in the analysis (Fig. 1).

### 2.2. Measurement of serum sodium concentration

Blood samples are collected and processed by certified laboratory professionals at a Mobile Examination Center, and then stored in a biological bank. As part of the conventional biochemical spectrum, serum chloride concentrations were measured using Beckman Synchron LX20 or Beckman Coulter UniCel DxC800 (Beckman Coulter, Inc., Brea), both of which used indirect (or diluted) ion selective electrode methods to determine serum sodium concentrations. The NHANES website provides detailed information about laboratory procedures (https://wwwn.cdc.gov/Nchs/Data/Nhanes/Public/).

### 2.3. The assessment of diet sodium level

The NHANES collected dietary intake information from participants through two 24-hour dietary recall interviews. The first one was done in person in the mobile examination center, and the second one was completed by phone between 3 and 10 days later. The United States Department of Agriculture’s Food and Nutrient Database for Dietary Studies was employed to calculate dietary sodium intakes. In this study, the diet sodium level was determined by averaging the results of two 24-hour interviews.

### 2.4. Definition of hypertension

In the current study, the outcome variable under investigation was hypertension, which was defined as the self-report hypertension, or systolic BP ≥ 140 mm Hg, or diastolic BP ≥ 90 mm Hg, or taking antihypertensive drugs.

### 2.5. Covariates

Through surveys and lab tests, baseline data on participants’ ages, sexes, educational levels, race, and body mass index (BMI) were gathered. BMI was computed by measuring the height and weight. The ratio of family income to poverty, which is the ratio of family income divided by a set poverty criterion for family size, was used to calculate income. We divided race into White, Black, Mexican, and other groups. Educational background was divided into less than high school, high school (or equivalent), and more than high school. Smoking status was classified as never smokers (smoke <100 cigarettes in their lifetime), current smokers (smoke <100 cigarettes, or had smoked some days), and former smokers (smoke at least 100 cigarettes and had quit smoking). The diagnostic criteria for alcohol consumption and status were heavy drinking (≥4 drinks/d for men, ≥3 drinks/d for women, or binge drinking ≥5 d/mo), moderate drinking (≥3 drinks/d for men, ≥2 drinks/d for women, or binge drinking ≥2 d/mo), mild drinking (not meeting the above criteria), never (had <12 drinks in lifetime), or former (had ≥12 drinks in 1 year and did not drink last year, or did not drink last year but drank ≥12 drinks in lifetime). Physical activity was evaluated by self-reported and measured in weekly metabolic equivalent (MET). MET < 600 was considered inactive, <600 was considered active, participants were categorized as inactive and active. The estimated glomerular filtration rate (eGFR) was calculated using the chronic kidney disease Epidemiology Collaboration algorithm. Self-reported questionnaires were used to collect information on the prevalence of CVDs, including myocardial infarction, coronary revascularization or stroke.

### 2.6. Statistical analysis

In this study, the data descriptions and statistical analyses were complexly weighted using the “Survey” package in R software (version 4.4.1). Continuous variables were expressed as the survey-weighted mean and standard errors. Survey-weighted *t* test was employed to assess the differences between 2 groups for continuous variables of normal distribution. Categorical variables were presented as absolute values (n) and weighted proportions (%), and were analyzed using the survey-weighted chi-square test.

In present study, weighted logistic regression analysis with 2 models were used to evaluate the odds ratios (OR) and 95% CIs between serum and diet sodium level and hypertension risk. Model 1 adjusted for age and sex; model 2 was based on model 1 and included additional factors such as ratio of family income to poverty, ethnicity, BMI, alcohol and smoke status, physical activity, total energy intake, eGFR, and CVDs. We investigated the serum and dietary sodium level by examining it as a categorical variable (Q1, Q2, Q3, and Q4). The trend test was used to detect the linear trend in weighted logistic regression. Additionally, we computed variance inflation factors to assess the presence of multicollinearity among the independent variables within the multiple regression model. A criterion of variance inflation factors <5 was employed to evaluate the independence of the variables in terms of collinearity.

Furthermore, weighted restricted cubic spline logistic regressions were used to examine the potential nonlinear dose–response associations between serum and dietary sodium level and the risk of hypertension in model 2. Our study used 3 knots (10th, 50th, and 90th percentiles), which is a standard practice to avoid model overfitting caused by an excessive number of knots. A stratified analysis was carried out according to the stratified variables such as age, sex, BMI, physical activity level, smoking status, alcohol consumption, and the presence or absence of CVDs using the fully adjusted model except for the specific stratification variable. The likelihood ratio test also inspected interactions of dietary and serum sodium with the stratification variables. In this study, a 2-side *P* < .05 was considered statistically significant.

## 3. Results

### 3.1. Baseline characteristics of participants

As shown in Table 1, the mean age of the study population was 47.53 ± 0.33 years, and the average BMI was 29.36 ± 0.12 kg/m^2^. Males accounted for 48.11% of the population, and 67.81% of the participants were white. According to the diagnostic criteria, 5763 participants were diagnosed with hypertension, and the weighted prevalence of hypertension was 38.14%. Table 1 presents a summary of the baseline characteristics of the included individuals, stratified by the presence or absence of hypertension. We observed significant differences in age, ethnicity, sex, BMI, alcohol consumption, smoking status, physical activity level, and total energy intake between the 2 groups (*P* < .01). Specifically, hypertensive participants were more likely to be older, have a higher BMI, and have a lower eGFR.

**Table 1:** Characteristics of study population.

Variable	Total (N = 15,349)	No (N = 9586)	Yes (N = 5763)	*P* value
Age (yr)	47.53 ± 0.33	41.31 ± 0.36	57.61 ± 0.31	<.01
Sex, n (%)				.03
Male	7402 (48.11)	4223 (47.34)	3179 (49.35)	
Female	7947 (51.89)	4669 (52.66)	3278 (50.65)	
Ethnicity, n (%)				<.01
White	6082 (67.81)	3430 (66.39)	2652 (70.10)	
Black	3410 (10.49)	1630 (9.04)	1780 (12.85)	
Mexican	2087 (8.21)	1381 (9.72)	706 (5.75)	
Other	3770 (13.50)	2451 (14.85)	1319 (11.30)	
Education, n (%)				<.01
Less than high school	3023 (12.78)	1573 (11.63)	1450 (14.65)	
High school	3548 (22.82)	1974 (21.62)	1574 (24.76)	
More than high school	8778 (64.40)	5345 (66.74)	3433 (60.59)	
BMI (kg/m^2^)	29.36 ± 0.12	28.14 ± 0.13	31.34 ± 0.14	<.01
PIR	3.05 ± 0.05	3.07 ± 0.06	3.02 ± 0.05	.17
Alcohol user, n (%)				<.01
Never	2375 (11.35)	1412 (11.39)	963 (11.28)	
Former	2052 (11.10)	886 (8.65)	1166 (15.09)	
Mild	5513 (38.85)	3090 (37.70)	2423 (40.72)	
Moderate	2480 (18.35)	1577 (19.87)	903 (15.88)	
Heavy	2929 (20.35)	1927 (22.39)	1002 (17.03)	
Smoking, n (%)				<.01
Never	9003 (57.87)	5665 (62.18)	3338 (50.87)	
Former	3553 (24.83)	1610 (20.58)	1943 (31.72)	
Now	2793 (17.30)	1617 (17.24)	1176 (17.41)	
Dietary sodium level (mg/d)	3370.80 ± 18.81	3401.36 ± 22.79	3321.24 ± 28.49	.02
Serum sodium level (mmol/L)	139.34 ± 0.11	139.38 ± 0.10	139.28 ± 0.13	.11
eGFR (mL/min)	94.45 ± 0.39	100.56 ± 0.45	84.54 ± 0.42	<.01
Physical activity, n(%)				<.01
Inactive	5821 (33.82)	2805 (27.89)	3016 (43.43)	
Active	9528 (66.18)	6087 (72.11)	3441 (56.57)	
CVD, n (%)				<.01
No	13,182 (89.23)	8015 (96.56)	5167 (82.99)	
Yes	1583 (8.52)	324 (3.44)	1259 (17.01)	

Continuous variables were expressed as the survey-weighted mean and standard errors; Categorical variables were presented as absolute values (n) and weighted proportions (%).

Chi-square tests for categorical variables and *t* test for continuous variables.

BMI = body mass index, CVDs = cardiovascular diseases, eGFR = estimated glomerular filtration rate, PIR = ratio of family income to poverty.

### 3.2. Association between dietary sodium and hypertension

**Figure 2.**
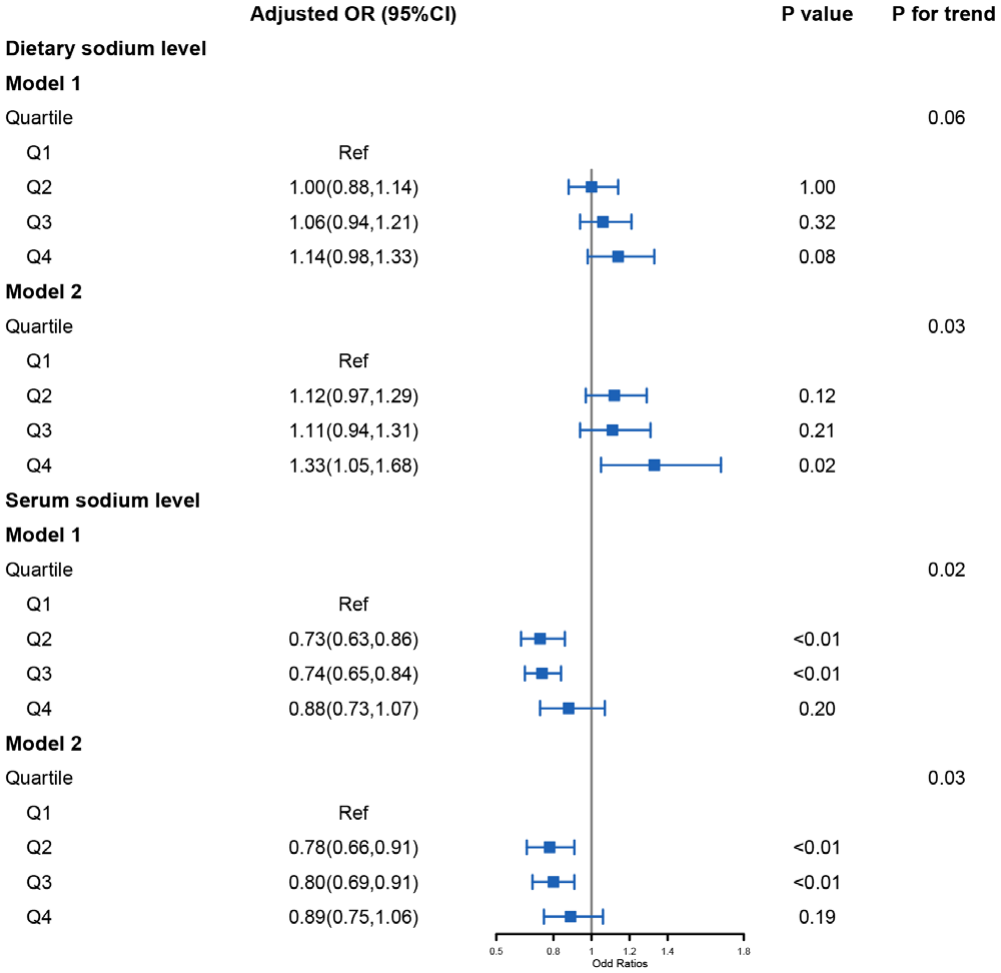
Weighted logistic regression analysis of the association between dietary and serum sodium and hypertension risk. Model 1: adjusted for age and sex; model 2: model 2 was based on model 1 and plus ethnicity, PIR, alcohol and smoke status, physical activity, total energy intake, eGFR and CVDs. The distribution of dietary sodium level in each quartile is as follow: Q1 (0, 2122] (N = 3844), Q2 (2122, 2995] (N = 3833), Q3 (2995, 4118] (N = 3838) and Q4 (4118, 20,683] (N = 3834). The distribution of serum sodium level in each quartile is as follow: Q1 (119, 138] (N = 5208), Q2 (138, 139] (N = 2789), Q3 (139, 141] (N = 4790), and Q4 (141, 161] (N = 2562). CI = confidence interval, CVDs = cardiovascular diseases, OR = odds ratios, PIR = ratio of family income to poverty.

To explore the associations between dietary sodium levels and the risk of hypertension, dietary sodium levels were categorized into 4 groups based on quantiles. The dietary sodium intake was distributed as follows, Q1: (0, 2122], Q2: (2122, 2995], Q3: (2995, 4118], and Q4: (4118, 20,683]. The mean level in Q1 was 1538.20 ± 11.17 mg/d, in Q2 was 2563.01 ± 5.40 mg/d, in Q3 was 3512.92 ± 6.55 mg/d, and in Q4 was 5547.39 ± 29.78 mg/d. The results are displayed in Figure 2. In model 1, which was adjusted for age and sex, there was no significant correlation between dietary sodium levels and the risk of hypertension (*P* > .05, *P* for trend > .05). However, in model 2, on the basis of the factors adjusted in model 1, additional factors such as BMI, ethnicity, smoking and drinking status, physical activity level, eGFR, and the presence or absence of CVDs were further incorporated for adjustment. Then, the correlation between dietary sodium levels and the risk of hypertension became evident. When compared to patients in the first tertile, those in the highest quartile had a significantly elevated risk of hypertension, with a 1.33-fold increase (OR = 1.33, 95% CI: 1.05–1.68, *P* = .02, *P* for trend = .03).

### 3.3. Association between serum sodium and hypertension

In the current study, the serum sodium level was distributed as follow, Q1: (119, 138], Q2: (138, 139], Q3: (139, 141], and Q4: (141, 161]. The mean level in Q1 was 136.81 ± 0.04 mmol/L, in Q2 was 139.00 ± 0.00 mmol/L, in Q3 was 140.43 ± 0.01 mmol/L, and in Q4 was 142.91 ± 0.07 mmol/L. We utilized 2 models to assess the relationship between serum sodium level and the risk of hypertension. The results are illustrated in Figure 2. Notably, unlike dietary sodium, the serum sodium level was found to have an inverse association with the risk of hypertension.

In model 1, when compared to the first quartile, the risk of hypertension was marginally lower in the second quartile (OR = 0.73, 95% CI: 0.63–0.86, *P* < .01) and the third quartile (OR = 0.74, 95% CI: 0.65–0.84, *P* < .01), with a significant trend (*P* for trend = .02). In the highest quartile, although it exhibited a reduced risk of hypertension, it has no significance (OR = 0.88, 95% CI: 0.73–1.07, *P* = .20). In the fully adjusted model, patients in the second and third quartile had a significantly lower risk of hypertension compared to those in the first quartile. Specifically, there was an 22% reduction in the second quartile (OR = 0.78, 95% CI: 0.66–0.91, *P* < .01) and a 20% reduction in the highest quartile (OR = 0.80, 95% CI: 0.69–0.91, *P* < .01, *P* for trend = .03). However, participants in the highest quartile has not showed a significant association with hypertension risk (OR = 0.89, 95% CI: 0.75–1.06, *P* = .17).

### 3.4. Restricted cubic spline analysis of the relationship between diet and serum sodium levels and hypertension

**Figure 3.**
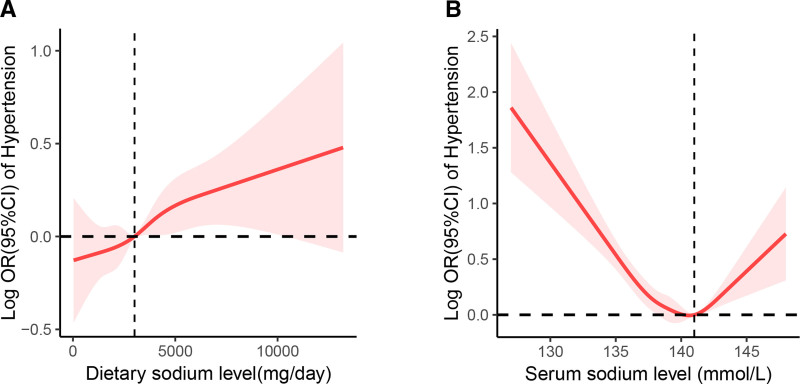
The dose–response relationship between dietary and serum sodium and the risk of hypertension. Dietary sodium was observed to linear positive correlated with hypertension (A), and serum sodium showed a “V” shape relationship with the hypertension risk (B). Adjusted for age, sex, ethnicity, PIR, alcohol and smoke status, physical activity, total energy intake, eGFR and CVDs. CVDs = cardiovascular diseases, eGFR = estimated glomerular filtration rate, PIR = ratio of family income to poverty.

The results of the restricted cubic spline analysis demonstrated that the dietary sodium level exhibited a linear positive association with the prevalence of hypertension (*P* for nonlinearity = .70; see Fig. 3A). In contrast, the serum sodium level showed a “V” shape relationship with the hypertension risk, indicating a nonlinear correlation with the risk of hypertension (*P* for nonlinear < .01; see Fig. 3B). Specifically, when the serum sodium level was lower than 141 mmol/L, the risk of hypertension decreased. Conversely, once the serum sodium level exceeded this value, the risk of hypertension increased.

### 3.5. Stratified analysis

Moreover, the stratified analyses were conducted. The results, presented in Table 2, demonstrated that the association between dietary sodium and hypertension varied across different age groups, genders, BMI categories, smoking and alcohol use statuses, physical activity levels and the presence or absence of CVDs. Notably, a positive association was specifically observed among older adults, females, overweight or obese individuals, never smoke and nondrinkers, those with low physical activity, and those without CVDs (*P* for trend < .05, Table 2). Additionally, we identified an interaction between different age groups and BMI groups regarding the correlation between dietary sodium intake and the risk of hypertension (*P* for interaction < .05, Table 2).

**Table 2:** Stratified analysis of the association between dietary and serum sodium and hypertension risk.

Character	Quartiles of dietary sodium level				*P* for trend	*P* for interaction
	Q1 (N = 3844)	Q2 (N = 3833)	Q3 (N = 3838)	Q4 (N = 3834)		
Age group						.01
<45 yr	Ref	1.56 (1.18, 2.06)	1.42 (1.05, 1.92)	1.50 (1.03, 2.17)	.19	
>45 yr	Ref	1.03 (0.85, 1.25)	1.04 (00.86, 1.27)	1.33 (1.02, 1.72)	.03	
Sex						.43
Male	Ref	1.22 (0.93, 1.62)	1.04 (0.81, 1.34)	1.20 (0.88, 1.61)	.46	
Female	Ref	1.11 (0.89, 1.38)	1.25 (0.98, 1.60)	1.59 (1.11, 2.28)	.01	
BMI group						.01
Normal	Ref	1.03 (0.76 1.40)	1.01 (0.68, 1.48)	0.97 (0.55, 1.69)	.88	
Overweight/Obese	Ref	1.15 (0.97, 1.37)	1.13 (0.94, 1.36)	1.40 (1.12, 1.75)	.01	
Smoke						.43
Never	Ref	1.14 (0.92, 1.41)	1.08 (0.86, 1.35)	1.40 (1.04, 1.89)	.04	
Former	Ref	1.09 (0.76, 1.59)	1.02 (0.73, 1.42)	1.41 (0.91, 2.18)	.14	
Now	Ref	1.17 (0.84, 1.64)	1.36 (0.96, 1.92)	1.05 (0.64, 1.70)	.93	
Alcohol user						.17
Never	Ref	1.79 (1.18, 2.70)	2.17 (1.44, 3.27)	3.39 (1.77, 6.47)	<.01	
Former	Ref	0.90 (0.61, 1.32)	1.25 (0.78, 1.98)	1.01 (0.50, 2.06)	.87	
Mild	Ref	1.08 (0.82, 1.43)	0.97 (0.72, 1.30)	1.35 (0.91, 2.02)	.13	
Moderate	Ref	1.19 (0.83, 1.70)	0.93 (0.57, 1.50)	1.54 (0.87, 2.70)	.24	
Heavy	Ref	1.10 (0.79, 1.55)	1.21 (0.88, 1.68)	0.97 (0.66, 1.43)	.75	
Physical activity						.54
Inactive	Ref	1.17 (0.93, 1.47)	1.37 (1.08, 1.75)	1.86 (1.36, 2.56)	<.01	
Active	Ref	1.10 (0.90, 1.36)	0.99 (0.78, 1.24)	1.10 (0.79, 1.52)	.74	
CVD						.76
No	Ref	1.13 (0.97, 1.33)	1.11 (0.94, 1.32)	1.36 (1.08, 1.71)	.02	
Yes	Ref	1.13 (0.73, 1.74)	1.00 (0.61, 1.62)	0.83 (0.44, 1.56)	.43	

Character	Quartiles of serum sodium level				*P* for trend	*P* for interaction
	Q1 (N = 5208)	Q2 (N = 2789)	Q3 (N = 4790)	Q4 (N = 2562)		
Age group						.07
<45 yr	Ref	0.86 (0.65, 1.15)	1.03 (0.82, 1.28)	1.10 (0.81, 1.49)	.49	
>45 yr	Ref	0.70 (0.56, 0.89)	0.68 (0.56, 0.82)	0.84 (0.66, 1.08)	.02	
Sex						.16
Male	Ref	0.66 (0.54, 0.81)	0.79 (0.65, 0.96)	0.87 (0.67, 1.12)	.16	
Female	Ref	0.87 (0.68, 1.11)	0.74 (0.59, 0.93)	0.96 (0.76, 1.21)	.12	
BMI group						.1
Normal	Ref	0.62 (0.43, 0.89)	0.63 (0.45, 0.90)	0.99 (0.72, 1.35)	.38	
Overweight/obese	Ref	0.81 (0.68, 0.97)	0.83 (0.72, 0.95)	0.91 (0.74, 1.13)	.11	
Smoke						.24
Never	Ref	0.84 (0.67, 1.06)	0.71 (0.58, 0.88)	0.92 (0.74, 1.15)	.06	
Former	Ref	0.63 (0.46, 0.87)	0.91 (0.72, 1.16)	0.94 (0.65, 1.35)	.82	
Now	Ref	0.74 (0.57, 0.97)	0.78 (0.60, 1.01)	0.91 (0.65, 1.29)	.25	
Alcohol user						.79
Never	Ref	0.84 (0.54, 1.31)	0.76 (0.54, 1.07)	1.08 (0.77, 1.52)	.68	
Former	Ref	1.00 (0.70, 1.43)	0.90 (0.59, 1.39)	1.05 (0.74, 1.50)	.86	
Mild	Ref	0.83 (0.61, 1.13)	0.76 (0.61, 0.94)	0.93 (0.68, 1.26)	.22	
Moderate	Ref	0.59 (0.36, 0.95)	0.72 (0.53, 0.96)	0.87 (0.57, 1.32)	.22	
Heavy	Ref	0.62 (0.44, 0.88)	0.83 (0.60, 1.16)	0.86 (0.56, 1.33)	.42	
Physical activity						.76
Inactive	Ref	0.76 (0.58, 1.00)	0.75 (0.59, 0.94)	0.81 (0.61, 1.08)	.04	
Active	Ref	0.76 (0.63, 0.92)	0.80 (0.66, 0.96)	1.01 (0.80, 1.27)	.44	
CVD						.9
No	Ref	0.75 (0.64, 0.88)	0.78 (0.67, 0.90)	0.93 (0.78, 1.12)	.07	
Yes	Ref	0.84 (0.49, 1.44)	0.69 (0.43, 1.11)	0.88 (0.49, 1.55)	.35	

Adjusted for age, sex, ethnicity, PIR, alcohol and smoke status, physical activity, dietary energy intakes, eGFR and CVDs. The distribution of dietary sodium level in each quartile is as follow: Q1 (0, 2122], Q2 (2122, 2995], Q3 (2995, 4118], and Q4 (4118, 20,683]. The distribution of serum sodium level in each quartile is as follow: Q1 (119, 138], Q2 (138, 139], Q3 (139, 141], and Q4 (141, 161]. The dietary and serum sodium levels in Q1 were set as reference.

BMI = body mass index, CVDs = cardiovascular diseases, eGFR = estimated glomerular filtration rate, PIR = ratio of family income to poverty.

Regarding the inverse association between serum sodium and hypertension, it remained consistent across different sex categories, BMI groups, smoking and alcohol consumption statuses, and the presence or absence of CVDs. However, this inverse relationship was particularly pronounced among older participants who led a sedentary lifestyle (*P* for trend < .05, Table 2).

## 4. Discussion

The present study revealed contrasting effects of dietary sodium and serum sodium on the prevalence of hypertension within the general adult population of the United States. Specifically, individuals in the highest quartile of dietary sodium intake levels exhibited a positive correlation with an elevated risk of hypertension. Conversely, the serum sodium levels within the second and third quartiles were found to have an inverse relationship with the risk of hypertension. Our findings underscore the importance for individuals following a low-sodium diet to closely monitor their serum sodium levels. Those who have adopted a low-sodium dietary pattern should be vigilant about maintaining appropriate serum sodium levels. In particular, a low-sodium dietary and maintaining the serum sodium level at approximately 141 mmol/L may confer greater health advantages, potentially contributing to a reduced risk of hypertension and improved overall cardiovascular health.

Human cells need approximately 500 mg/d of sodium to sustain vital functions. According to the American Heart Association, the optimal daily sodium intake is 1500 mg/d, with a maximum recommended intake of 2300 mg/d.^[20]^ In this study, the average sodium intake of study population was 3400 mg/d (Table 1), more than double the ideal recommendations. Additionally, our results showed that, when compared to participants in the lowest quantile group, those in the second and third quantile groups did not exhibit a significant alteration in the risk of hypertension (*P* > .05). Conversely, subjects in the highest quantile group had a significantly elevated risk of hypertension (*P* < .05), thereby presenting a significant trend (*P* for trend = .02). Although the relationship between sodium intake, BP, and CVDs has been extensively investigated in both observational and experimental studies, these studies have consistently demonstrated that reducing dietary salt intake can lower BP.^[21]^ It was estimated that a 6000 mg/d increase in salt intake over 30 years would lead to a 9 mm Hg increase in systolic BP. Another meta-analysis of 12 cohort studies indicated that a 5000 mg/d increase in salt intake was associated with a 23% increase in the risk of stroke and a 17% increase in the risk of CVDs.^[22]^

Unlike other studies that reduced salt intake from a much higher level to a relatively lower 1, this study elucidated the distribution of dietary sodium intake levels in the general population. It revealed that a dietary sodium intake level between 1500 and 3500 mg/d had no significant impact on the risk of hypertension. When the sodium intake level increased to the highest quartile of dietary sodium intake levels, with a mean level of 5547.39 ± 29.78 mg/d, individuals had a 1.33-fold increase in the risk of hypertension after adjusting for all covariates (Fig. 2). Furthermore, stratified analysis revealed that this association was particularly notable among older adults, females, overweight or obese individuals, never smoke and nondrinkers, those with low physical activity, and those without CVDs (Table 2). This can be attributed to the phenomenon of “sodium sensitivity,” individual BP responses to dietary sodium intake vary significantly.^[23,24]^ Blacks, women, the elderly, and those with hypertension, obesity, or chronic kidney disease are more sensitive to dietary sodium intake.^[25,26]^

However, the mechanisms by which lower sodium decreases BP are not fully understood. There is now increasing evidence that small changes in plasma sodium may be an important mechanism for the changes in BP with changing salt intake. A number of studies have shown that an increase or a decrease in salt intake causes parallel changes in plasma sodium in both hypertensive and normotensive individuals.^[27,28]^ In a well controlled double-blind trial, plasma sodium was reduced by 0.4 mmol/L (*P* < .05) when salt intake was decreased from 10,000 mg/d to 5000 mg/d in 118 hypertensive individuals over 1 month. It has also been reported that serum sodium distribution patterns differed between normal subjects and patients with essential hypertension in a Japanese population.^[29]^ In this study, no significant difference was observed in the distribution of serum sodium in the study population grouped by the absence or presence of hypertension (Table 1). Additionally, there was no significant difference observed in the serum sodium level according to the quantiles of dietary sodium (*P* = .55, Table S1, Supplemental Digital Content, https://links.lww.com/MD/Q251). Furthermore, we observed that a serum sodium level within the range of 138 to 141 mmol/L was associated with a lower risk of hypertension. In contrast, both a too low and a too high serum sodium level might led to an increased risk of hypertension (Figs. 2 and 3). This can probably be ascribed to a 5% increase of plasma sodium concentration (sodium excess) stiffens endothelial cells by about 25%, leading to cellular dysfunction.^[30,31]^

A cohort study conducted in Japan aiming to elucidate the association between serum sodium concentration and the risk of incidence hypertension reported that compared with the lowest quartile of serum sodium level (137–140 mmol/L), the multivariable-adjusted hazard ratios (95% CI) for incident hypertension were 1.03 (0.71–1.51), 1.35 (0.87–2.08), and 1.46 (0.97–2.20) for the upper 3 quartiles of the serum sodium levels (Q2: 141–142 mmol/L; Q3: 143mmol/L; Q4: 144–147mmol/L), with a *P* for trend of .02.^[32]^ This results were consistent with our study and revealed that participants with a serum sodium level within 137 to 140 mmol/L (138–141 mmol/L in our study) had the lowest risk of hypertension. Although a positive correlation between serum sodium and the risk of hypertension has been reported, the differences in quartiles of serum sodium level should be carefully considered. The Framingham Heart Study, based on a community sample, reported that serum sodium was not associated with hypertension incidence.^[16]^ It reported that per SD of serum sodium level increased, the risk of hypertension incidence was decreased by 6% with an OR value of 0.94 (95% CI: 0.82–1.08) during 4 years of follow-up, although this was not significant (*P* > .05). These different results might be attributed the relatively small sample size, as a community sample was used in the Framingham heart study.

## 5. Strength and limitation

However, it is crucial to recognize several limitations of this study. Firstly, given the observational study design, a causal relationship cannot be established. Randomized controlled trials are necessary to confirm causality. Secondly, serum sodium levels can be influenced by short-term factors. Measuring serum sodium levels only once at baseline does not accurately represent an individual’s true long-term serum sodium statues. Therefore, in future research, repeated measurements are highly recommended. Third, the study participants were adult civilians in the United States. When extrapolating the results to other populations, the associations and effect sizes need to be further validated. Fourth, the method of handling missing values can also impact the research outcomes. In this study, we excluded subjects with missing values. Finally, the presence of residual and unknown confounding factors, such as the use of diuretics, cannot be entirely ruled out. Additionally, some information was obtained through self-reporting, which may be subject to bias.

Despite these limitations, this study also had several strengths. The relatively large sample size and the nationally representative sample design enhanced the generalizability of our findings. Furthermore, we controlled for a wide array of potential confounding factors, including sociodemographic factors, lifestyle factors, laboratory test results, and commodities, using comprehensive statistical methods. This comprehensive approach ensured the robustness of our research conclusions.

## 6. Conclusion

In conclusion, our analysis of the general US adult population has reveals a positive association between the highest quartile of dietary sodium intake and the risk of hypertension. This association is particularly pronounced among older adults, females, overweight or obese individuals, those who have never smoked and do not drink alcohol, those with low physical activity levels, and those without CVDs. Moreover, we have identified a “V”-shaped nonlinear relationship between serum sodium levels and the risk of hypertension. This inverse association is especially evident among older participants with a sedentary lifestyle. Further in-depth research is clearly warranted. Well-designed prospective cohort studies and interventional trials are essential to establish a causal relationship and to clarify the underlying mechanisms. Notably, adhering to a low-sodium diet and maintaining serum sodium levels at approximately 141 mmol/L may confer substantial health benefits. This approach has the potential to reduce the risk of hypertension and improve overall cardiovascular health.

## Author contributions

**Conceptualization:** Hongpeng Liu, Qing Wu.

**Data curation:** Jianmei Zhou, Shanshan Xing.

**Methodology:** Hongpeng Liu, Jianmei Zhou, Shanshan Xing.

**Visualization:** Qing Wu.

**Writing** – **original draft:** Hongpeng Liu, Qing Wu.

**Writing** – **review & editing:** Jianmei Zhou, Qing Wu, Shanshan Xing.

## Supplementary Material


