# Supplementary material for: The opposing association of diet and serum sodium with the prevalence of hypertension in the US adult general population: A cross-section study
Source: Medicine (Baltimore). 2025 Oct 24;104(43):e45103. doi: 10.1097/MD.0000000000045103 (PMC12558238; doi:10.1097/MD.0000000000045103)
Supplement: Supplementary file 1 [file medi-104-e45103-s001.docx]

Table S1 The distribution of serum sodium level according to the quantiles of dietary sodium

| **Variable** | **Total (N=15,349)** | **Quartiles of Dietary Sodium** | | | | **p value** |
| --- | --- | --- | --- | --- | --- | --- |
|  |  | **Q1** | **Q2** | **Q3** | **Q4** |  |
| **Dietary sodium (mg/day)** | 3370.80±18.81 | 1538.20±11.17 | 2563.01±5.40 | 3512.92±6.55 | 5547.39±29.78 | <0.01 |
| **Serum sodium (mmol/L)** | 139.34±0.11 | 139.38±0.12 | 139.37±0.12 | 139.33±0.11 | 139.29±0.12 | 0.55 |

Variables were expressed as the survey-weighted mean and standard errors, ANOVA analysis was used to calculate the p value. The distribution of dietary sodium level in each quartile is as follow: Q1 (0, 2122] (N=3844), Q2 (2122, 2995] (N=3833), Q3 (2995, 4118] (N=3838) and Q4 (4118, 20683] (N=3834). The distribution of serum sodium level in each quartile is as follow: Q1 (119, 138] (N=5208), Q2 (138, 139] (N=2789), Q3 (139, 141] (N=4790), and Q4 (141, 161] (N=2562).
